# Low-threshold nanolasers based on miniaturized bound states in the continuum

**DOI:** 10.1126/sciadv.ade8817

**Published:** 2022-12-23

**Authors:** Yuhao Ren, Peishen Li, Zhuojun Liu, Zihao Chen, You-Ling Chen, Chao Peng, Jin Liu

**Affiliations:** ^1^ State Key Laboratory of Optoelectronic Materials and Technologies, School of Physics, Sun Yat-sen University, Guangzhou 510275, China.; ^2^ State Key Laboratory of Advanced Optical Communication System and Networks, School of Electronics and Frontiers Science Center for Nano-optoelectronics, Peking University, Beijing 100871, China.; ^3^ State Key Laboratory for Mesoscopic Physics and Frontiers Science Center for Nano-optoelectronics, School of Physics, Peking University, Beijing 100871, China.; ^4^ State Key Laboratory of Integrated Optoelectronics, Institute of Semiconductors, Chinese Academy of Sciences, Beijing 100083, China.; ^5^ Peng Cheng Laboratory, Shenzhen 518055, China.

## Abstract

The pursuit of compact lasers with low thresholds has imposed strict requirements on tight light confinements with minimized radiation losses. Bound states in the continuum (BICs) have been recently demonstrated as an effective mechanism to trap light. However, most reported BIC lasers are still bulky due to the absence of in-plane light confinement. Here, we combine BICs and photonic bandgaps to realize three-dimensional light confinements, as referred to miniaturized BICs (mini-BICs). We demonstrate highly compact active mini-BIC resonators with a record high-quality (*Q*) factor of up to 32,500, which enables single-mode lasing with the lowest threshold of 80 W/cm^2^ among the reported BIC lasers. In addition, photon statistics measurements further confirm the occurrence of the stimulated emission in our devices. Our work reveals a path toward compact BIC lasers with ultralow power consumption and potentially boosts the applications in cavity quantum electrodynamics, nonlinear optics, and integrated photonics.

## Teaser

Miniaturized photonic bound states in the continuum facilitate the realization of low-threshold single-mode nanolasers.

## INTRODUCTION

Nanoscale coherent light generations via stimulated emissions have been the scientific frontier of nanophononics, topological photonics (*1*–*3*), non-Hermitian physics (*4*–*6*), and optics in random media (*7*, *8*). From the viewpoint of technology, the scalable creations of miniaturized lasers with low-power consumption enable a variety of important applications across optical interconnects (*9*, *10*), biosensing (*11*), and far-field beam synthesis (*12*, *13*). To achieve lasing at the extreme subwavelength scale, plasmonic cavities are usually used; however, they unavoidably suffer from high ohmic losses associated with metals (*14*, *15*). While at the wavelength scale, dielectric nanolasers have been realized with the assistance of high-quality (*Q*) cavities using total internal reflection or photonic bandgaps (PBGs) (*16*–*19*), such as microdisks or photonic crystal (PhC) defect cavities. However, because of the limited lasing volume, their emission power is still not quite sufficient in driving the applications, for instance, on-chip optical communications. Recently, several designs such as random laser (*20*, *21*), topological laser (*22*, *23*), and moiré lattice laser (*24*) had been proposed to achieve lasing behavior at a 10-wavelength scale to best compromise the footprint and power.

Trapping light is no doubt the first step toward nanoscale lasers. As an emerging mechanism, bound states in the continuum (BICs) have been demonstrated as a very powerful tool to suppress out-of-plane radiations and consequently boost the *Q* factors of planar optical resonators (*25*, *26*). In addition, the vectorial nature of BICs enables emissions of structured light from chip-scale devices, leading to ultrafast switchable nanolasers (*27*) and multiplexed nanolasers carrying orbital angular momenta (*28*). In principle, ideal BICs with infinite *Q*s only exist in periodic and symmetric structures. Therefore, early demonstrations of BICs used relatively large sample sizes ranging from a few tens to hundreds of periodic unit cell to maintain the high-*Q* feature (*29*, *30*). One of the successful efforts in promoting the *Q*s is to topologically merge a set of BICs into the so-called super-BIC regime (*31*), which markedly minimizes the radiation loss and therefore substantially reduces the thresholds of BIC lasers (*32*).

To make nanolasers suitable for practical applications with higher output powers, we look for the BICs in miniaturized sizes, namely, a 10-wavelength scale. For active devices, the benefits of shrinking the mode sizes lies on two folds: First, in the spontaneous emission regime, the small mode volumes can greatly enhance the strength of light-matter interactions at a single-photon level, enabling the explorations of cavity quantum electrodynamics (QED) effects in both weak and strong coupling regimes (*33*); second, in the stimulated emission regime, miniaturized mode volumes produce strong light trapping that significantly reduces the lasing thresholds (*34*). However, the symmetry breaking or truncating the infinite size, transiting ideal BICs to quasi ones (*35*, *36*), unavoidably lowers down the *Q* factors accordingly and makes them less favorable for low-threshold lasing. Thus, it is highly desirable yet an on-going challenge to achieve miniaturized BICs (mini-BICs) with high-*Q* factors.

In this work, we simultaneously use the BICs to suppress the out-of-plane radiation and use the PBGs to minimize the in-plane optical dissipations for achieving high-*Q* optical resonators with small footprints. We fabricated active GaAs membranes supporting mini-BICs with *Q*s as high as ∼32,500 and exploited high-density InAs quantum dots (QDs) as optical gain materials. Laser oscillations at telecom O band under both continuous wave (CW) and pulsed optical pumping were observed with a threshold down to 80 W/cm^2^, which is nearly two orders of magnitude lower than the previous reported BIC lasers (*32*). We systematically compare the lasing and nonlasing behaviors by using time-resolved and photon statistics measurements, further revealing the phase transition from spontaneous to stimulated emission in our devices. This work serves as a crucial step toward understanding and realizing high-performance BIC lasers with miniaturized footprints and may further boost the applications of BICs in cavity QED and integrated nonlinear photonics, which demand for both high-*Q* and low mode volume cavities.

## RESULTS

**Fig. 1.**
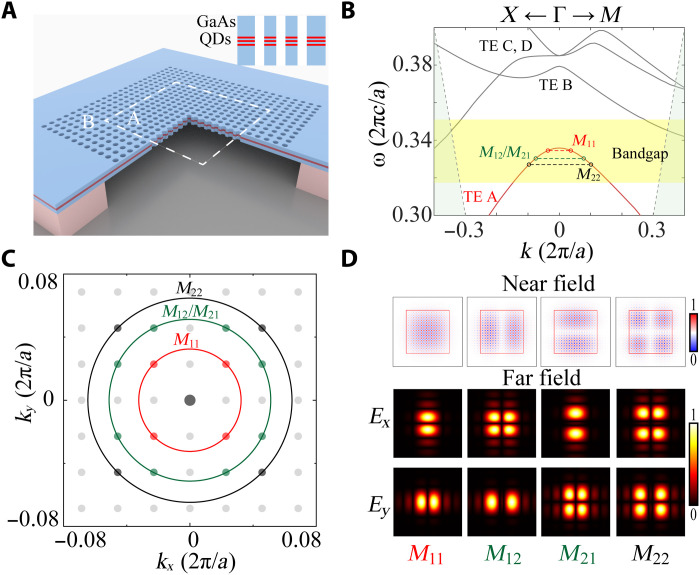
Design of mini-BIC lasers. (**A**) Schematic of mini-BIC laser device consisting of a suspended GaAs thin membrane with periodically etched air holes. The PhCs in regime A is surrounded by other heterogeneous photonic crystal (PhCs) in region B. Three layers of QDs are embedded in the center of the membrane as optical gain materials. Inset: Cross section of the etched membrane with three layers of QDs. (**B**) The band diagrams of PhCs in regime A (with infinite size). The continuous band (TE-A; represented by the red line) associated with ideal PhCs in regime A was quantized into discrete modes above the light line and located in the bandgap of PhCs in region B (represented by the yellow area). (**C**) The momentum distribution of each mode, modes are labeled as *M*_pq_, according to their momentum peak positions in the first quadrant. (**D**) The near-field (6 μm by 6 μm) and far-field patterns (30° by 30°) of four modes *M*_11_ to *M*_22_.

Our laser cavity is based on PhCs consisting of a suspended GaAs membrane with periodically etched air holes in a square lattice array, as schematically shown in Fig. 1A. Three layers of high-density (10^10^cm^-2^) InAs QDs are embedded in the center of GaAs membranes as optical gain materials at telecom O band. The details of the QD epitaxial wafer are presented in fig. S1. To tightly localize the light in three dimensions, we explored a recent proposal of mini-BICs in which the out-of-plane radiation is suppressed by BICs, while the in-plane light confinement is achieved by PBGs (*37*). The BICs associated with the PhCs in region A (lattice constant *a*) is surrounded by heterogeneous PhCs in region B (lattice constant *b*) with a gap size of *g* in between. The band diagram of the designed structure is shown in Fig. 1B, in which the transverse electric mode A (TE-A) mode of PhCs in region A sits in the bandgap of PhCs in region B. Therefore, region B serves as highly reflective mirrors to suppress the in-plane light leakage from the mini-BICs residing in region A and, thus, significantly improves the *Q* factor and reduces mode volume *V*. In addition, the finite size of the cavity region A quantizes the continuous TE-A band into discrete modes with a mode spacing of δ*k* = π/*L*, where *L* is the cavity length of region A. Each mode can thus be labeled by a pair of integers (*p*, *q*), indicating that its momentum is mostly localized near *p*π/*L*; *q*π/*L* in the first quadrant of the momentum space. In Fig. 1C, we plot the highly momentum-localized modes of *M*_11_, *M*_12_/*M*_21_, and *M*_22_ in the first quadrant of momentum space, in which *M*_12_ and *M*_21_ are degenerated in frequency because of the 90° rotation symmetry of the structure (*C*4). The near-field mode distributions reveal that the mini-BICs are highly spatially localized in the cavity region enclosed by the PBG mirrors, while such momentum space localizations result in highly directional emissions toward specific angles, as presented in Fig. 1D.

**Fig. 2.**
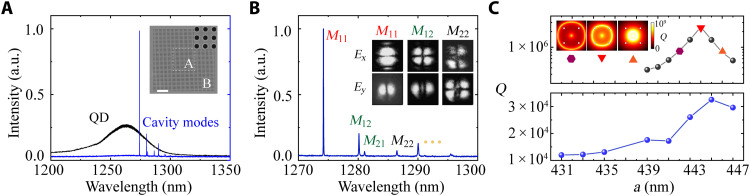
Optical characterizations of the active mini-BIC cavity. (**A**) Microphotoluminescence (μ-PL) spectra of QD ensemble (black) and the cavity modes (blue) of the mini-BIC. Inset: Top-view scanning electron microscopy image of the fabricated mini-BIC device. a.u., arbitrary units. (**B**) Zoomed-in μ-PL spectrum of the cavity modes associated with mini-BICs. The inset shows experimental far-field patterns (*x*/*y*-polarized) of modes *M*_11_ to *M*_22_. (**C**) Simulated (top) and measured (bottom) quality (*Q*) factors of the *M*_11_ mode as a function of the lattice constant *a*. Inset: High-*Q* rings corresponding to the constellation of multiple BICs in momentum space for three different lattice constants.

Experimentally, the mini-BIC patterns were defined on the electron beam (E-beam) resist using a 100-kV E-beam writer and then transferred to the GaAs layer via a chloride-based dry etch process. The GaAs membranes were released by selectively wet etching a 1500-nm-thick AlGaAs sacrificial layer underneath. The full fabrication flow of the devices is presented in fig. S2. Sharp resonances corresponding to the cavity modes were identified by measuring microphotoluminescence (μ-PL) from the cavity area at room temperature. The details of the homemade μ-PL setup are presented in figs. S3 and S4. For comparison, the μ-PL of the QD ensemble, indicating the optical gain spectrum, was obtained from the area without any cavity structures. As presented in Fig. 2A, by choosing a proper lattice constant *a* = 445 nm with 17 periodicities, *b* = 463 nm with 15 periodicities, and *g* = 455 nm, cavity modes are spectrally aligned with the gain spectrum to facilitate the lasing oscillations. The sharp resonances measured from the cavity area were further zoomed in Fig. 2B, where the optical characteristics such as resonant wavelength and mode spacing agreed very well with the simulations in Fig. 1. In addition, we performed the far-field characterizations of each mode (insets in Fig. 2B), further revealing the vectorial nature and momentum localization of the mini-BIC modes, as predicted in the Fig. 1D. The cavity resonances as a function of the PhC lattice constant *a* are presented in fig. S5, indicating that the measured cavity modes are associated to BICs in the PhCs. The dependence of the *Q* factor (measured at the transparent excitation power) on the constant *a* of the *M*_11_ mode is presented in Fig. 2C. As shown in the simulations, the *Q*s of mini-BIC can be engineered by tailoring the topological charges of the BICs via tuning the latticed constant of etched air holes.

**Fig. 3.**
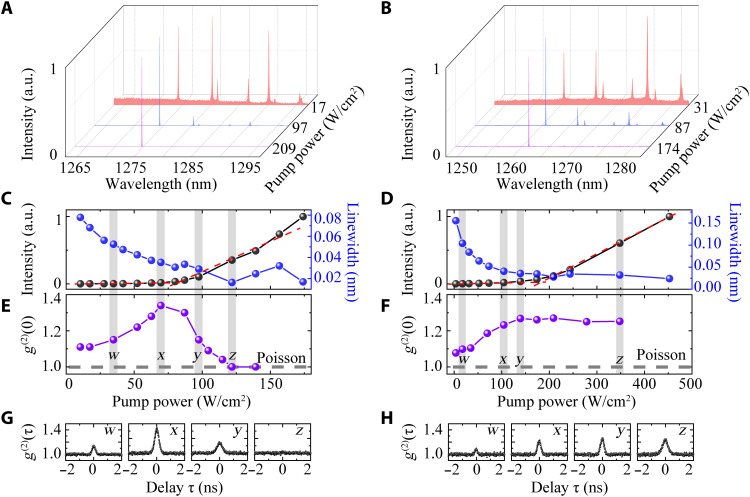
Characterizations of lasing and nonlasing behavior under CW excitations. (**A** and **B**) Evolution of the normalized emission spectra of the lasing device (A) and nonlasing device (B) with the increased excitation power. (**C** and **D**) Integrated output intensity and linewidth of the cavity mode *M*_11_ as a function of the excitation power, showing “threshold-like” behaviors at 80 W/cm^2^ (C) and 184 W/cm^2^ (D), respectively. The red dashed line shows different slopes of the output power when increasing the excitation power. The shaded areas represent the excitation powers at which the *g*^2^ trace are presented. (**E** and **F**) Second-order correlation functions at zero delay time *g*^2^(0) as a function of the excitation power. The lasing device exhibited a clear phase transition from spontaneous emission [a thermal state with *g*^2^(0) > 1] to stimulated emission [a coherent state with *g*^2^(0) = 1], while the nonlasing emissions remained in a thermal state for all the excitation powers. (**G** and **H**) Autocorrelation traces taken at the pump power densities marked in (C) and (D).

We measured the μ-PL spectra of one cavity under CW excitation with different excitation powers, as presented in Fig. 3A. Sharp cavity modes on a broad emission background can be identified under low excitation powers. By increasing the excitation power, *M*_11_ mode dominated the emission spectrum, and its linewidth reduced significantly. The strong suppression of other cavity modes under high excitation powers is due to the mode competition in the lasing process. The intensity and linewidth of the cavity mode *M*_11_ as a function of the excitation power were plotted in Fig. 3C. A sharp increase in the input-output (IO) curve together with reduction of the cavity linewidth, commonly believed as signatures of lasing, was observed, suggesting the occurrence of lasing oscillation.

To reliably identify the lasing oscillation, we characterized photon statistics of the emitted photons by measuring the second-order coherence (*38*, *39*), which can rigorously quantify the quantum nature of the phase transition from spontaneous emission to stimulated emission. In Fig. 3E, at very low excitation powers, the emission was in a thermal state but only exhibited a slight bunching behavior in *g*^2^(0) because of the relatively short coherence time of the emitted photons. With further increase of the excitation powers, the coherence time had significantly prolonged, and therefore, appreciable photon bunching effects were observed. As long as the excitation power crossed the lasing threshold of 41 μW with a beam diameter of 8.11 μm (see fig. S4), corresponding to a power density of 80 W/cm^2^, the thermal state evolved toward a coherent state, and the *g*^2^(0) gradually lowered down to 1 when far above threshold. The photon statistics evidence together with the IO curve and linewidth reduction unequivocally demonstrated the realization of lasing oscillation in our mini-BIC devices.

On the other hand, there have been increasing debates on using the kink feature upon IO curves as the single criterion of nanoscale lasing, especially when the device experiences cavity QED effects (*40*–*42*). To that end, we characterized a less-optimal mini-BIC device similarly and presented the experimental data in Fig. 3 (B, D, F, and H). The main difference between the lasing and less optimal device (nonlasing) is the spectral alignment between the cavity modes and gain media (see more details in fig. S6). This nonlasing device also exhibited a “threshold” feature upon the IO curve and a linewidth narrowing behavior, which are very similar to the lasing device shown in Fig. 3 (A and C). However, no signatures of phase transition from thermal emission to a coherent state were observed from the photon statistics measurements. As shown in Fig. 3F, the *g*^2^(0) raised monotonously with the increase of the excitation power, which indicates that the device was operating as a nano–light-emitting diode (nano-LED) instead of a nanolaser.

It is expected that the nonlasing device can also exhibit nonlinear output intensities upon the excitation power. Such a fact has been observed in a coupled single QD cavity system without involving lasing oscillations, as a consequence of the nonresonant couplings between different excitonic states of QDs and the cavity mode (*43*). At low excitation powers, the intensity of the cavity mode followed the linearly increased emission of exciton state formed in the singe QD. Under high excitation powers, the cavity mode showed a superlinear power dependence because of the nonresonant couplings to the biexciton states whose intensities grow nearly as twice as the exciton states. Such a mechanism could also be responsible for the nonlinear output intensities of the many QD-cavity–coupled system, as observed in Fig. 3D. The linewidth narrowing shown in Fig. 3D is easier to be understood, because the active cavity experienced more absorption from the gain material under low excitation powers. With the increase of excitation power, the excited states of QDs get populated, resulting in the reduction of the cavity linewidth.

**Fig. 4.**
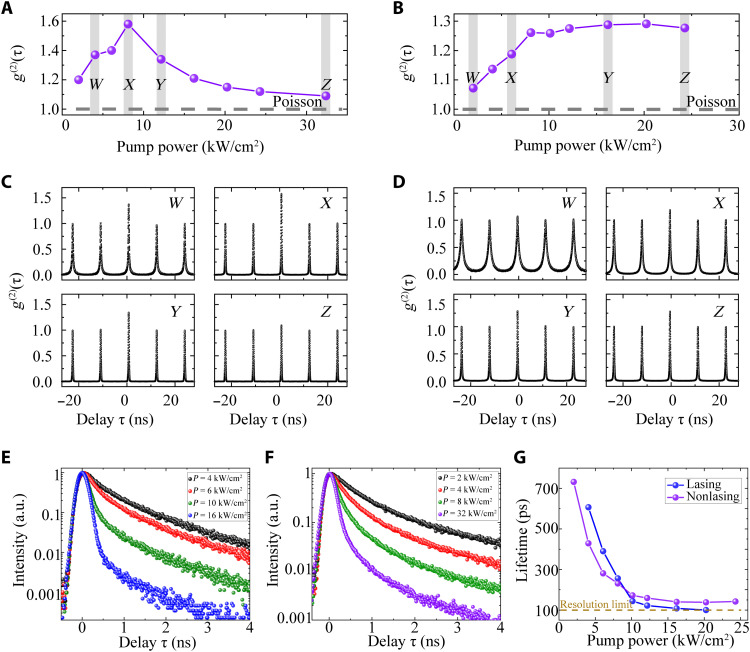
Characterizations of lasing and nonlasing devices under pulsed excitation. (**A** and **B**) Second-order correlation function at zero delay time *g*^2^(0) as a function of the excitation power. The lasing device exhibited a clear phase transition from spontaneous emission [a thermal state with *g*^2^(0) > 1] to stimulated emission [a coherent state with *g*^2^(0) =1] (A). The nonlasing device remained in a thermal state [*g*^2^(0) > 1] through all the excitation powers (B). The shaded areas *W*, *X*, *Y*, and *Z* represent the excitation powers at which the *g*^2^ trace are presented. (**C** and **D**) Autocorrelation traces taken at the pump power densities marked in (A) and (B). (**E** and **F**)The photon lifetime traces of lasing and nonlasing devices under different excitation powers. (**G**) The lifetimes of lasing and nonlasing devices as a function of the excitation power. The orange dashed line indicates the time resolution of our measurement system.

To investigate the possibility of turning the nonlasing behavior to a lasing oscillation with higher peak powers and less thermal effects, we further performed pulsed excitation on the same devices. The CW lasing device also exhibited clear a nonlinear IO curve and linewidth reductions, as presented in fig. S7. The pulsed *g*^2^(0) clear exhibited the phase transition from a thermal state to a coherent state when increasing the excitation power, as shown in Fig. 4 (A and C). On the contrary, the nano-LED device showed “laser-like” behaviors in terms of nonlinear increase in the IO curve and linewidth reductions, but its photon statistics remained in a thermal state across all the excitation power, as shown in Fig. 4 (B and D). One of the advantages of pulsed excitation scheme is that we are able to directly measure the photon lifetime (*44*), which can serve as additional evidence to distinct the fast stimulated emissions from the relatively slow spontaneous emission. The decay curves under different excitation powers for the laser and LED devices are presented in Fig. 4 (E and F), respectively. Their emissions lifetimes were shortened during the increasing of pulse power, as quantitatively presented in Fig. 4G. For the lasing device, the lifetime reduced more rapidly than those of the nano-LED until the measurement was beyond the time resolution of our photon detectors. Such fast decay rates above threshold are strong indications of stimulated emissions. The lifetime of photons emitted by the nano-LED device also decreased, which is expected because of the different carrier relaxation dynamics under high excitation powers (*45*). However, the photon lifetime of nano-LED reduced rather slowly as compared to the lasing device and became saturated (not reaching the time resolution limit of our measurement) at high excitation powers, which is more preferably to ascribe to spontaneous emission.

## DISCUSSION

We have demonstrated III-V semiconductor active mini-BIC resonators with a quality factor as high as ∼32,500, which enables the realizations of lasing oscillations with a record-low threshold of 80 W/cm^2^ that is nearly two orders of magnitude lower than the state-of-the-art BIC lasers (see table S1 for comparison). Both CW and pulsed lasing were unequivocally realized by systematically measuring the device characteristics including μ-PL spectra, IO curves, emission linewidth, photon statistics, and photon lifetimes. Our investigations suggest that any claim of nanolasing with cavity QED effects should be very careful and further comparisons between more advanced theory and experiments are indispensable. Moving forward, the vectorial nature of mini-BICs could be exploited to build chip-scale lasers capable of emitting coherent structured light for high-capacity optical communication (*46*) and high-dimension quantum information processing (*47*). It is also highly desirable to implement the electrical injections in active BIC devices, as successfully demonstrated for suspended PhC defect lasers (*48*) and on-substrate surface emitting laser with less thermal effect (*49*, *50*). From the perspective of applications, the realization of mini-BIC laser may immediately boost the development of on-chip cavity QED (*51*) and the integrated nonlinear photonics (*52*) in which high-Q factors and small mode volumes are highly beneficial.

## MATERIALS AND METHODS

### Device fabrication

The samples are fabricated using a 500-nm-thick GaAs/1.5-μm-thick Al_0.8_Ga_0.2_As/GaAs substrate wafer. Three layers of InAs/GaAs QDs are embedded in the middle of the first GaAs layer, whose central emission wavelength is 1.3 μm. The AlGaAs layer act as sacrificial and etch stop layer. First, a layer of Si_3_N_4_ with a thickness of 200 nm was deposited on the wafer by inductively coupled plasma (ICP) chemical vapor deposition as a hard mask for PhC dry etching. A 400-nm ARP6200 E-beam resist was spin coated on the surface of the hard mask. Subsequently, the E-beam lithography was used to define the PhC pattern in ARP6200. The PhC pattern was transferred from resist into the hard mask layer using reactive ion etching (RIE). Afterward, the E-beam resist was removed by ICP with O_2_ plasma. Then, ICP etching was performed to obtain the air holes through the active layer. The residual Si_3_N_4_ hard mask was removed by RIE dry etching. Last, the sacrificial layer was wet etching by immersing the sample in 10% hydrofluoric acid solution for 1 min and 30 s to form an air region slab underneath the PhC membrane.

### Optical measurement

The fabricated mini-BIC devices were characterized with a customized μ-PL measurement system in a surface normal pump configuration. A 780-nm CW laser diode and pulse laser diode (10 ps, 86-MHz period) were used to optically pump the sample via a 50× objective lens with a numerical aperture of 0.65. The emission spectra were collected by the same objective and analyzed by a spectrometer (iHR550) with a resolution of 0.025 nm.

## Supplementary Materials

### This PDF file includes:

Sections S1 to S7 Figs. S1 to S7 Table S1
