# Supplementary material for: Low-threshold nanolasers based on miniaturized bound states in the continuum
Source: Sci Adv. 2022 Dec 23;8(51):eade8817. doi: 10.1126/sciadv.ade8817 (PMC9788758; doi:10.1126/sciadv.ade8817)
Supplement: Supplementary file 1 — Sections S1 to S7 Figs. S1 to S7 Table S1 [file sciadv.ade8817_sm.pdf]

Science Advances
AAAS

Supplementary Materials for

Low-threshold nanolasers based on miniaturized bound states in the continuum

Yuhao Ren *et al.*

Corresponding author: You-Ling Chen, ylchen@semi.ac.cn; Chao Peng, pengchao@pku.edu.cn

Sci. Adv. **8**, eade8817 (2022)
DOI: 10.1126/sciadv.ade8817

This PDF file includes:

Sections S1 to S7
Figs. S1 to S7
Table S1

I. QUANTUM DOT WAFER

The QD wafer was grown via a molecular beam epitaxy. As schematically shown in Fig. S1(a), 300 nm GaAs buffer layer was firstly grown on a 350-μm-thick GaAs substrate followed by a 1500 nm AlGaAs sacrificial layer. 500 nm GaAs with three layers of high-density InAs QDs was finally grown on top. An atomic force microscope (AFM) image of uncapped InAs/GaAs QDs indicates a QD density of $\sim 10^{10}/\mathrm{cm}^2$ is presented in Fig. S1(b).

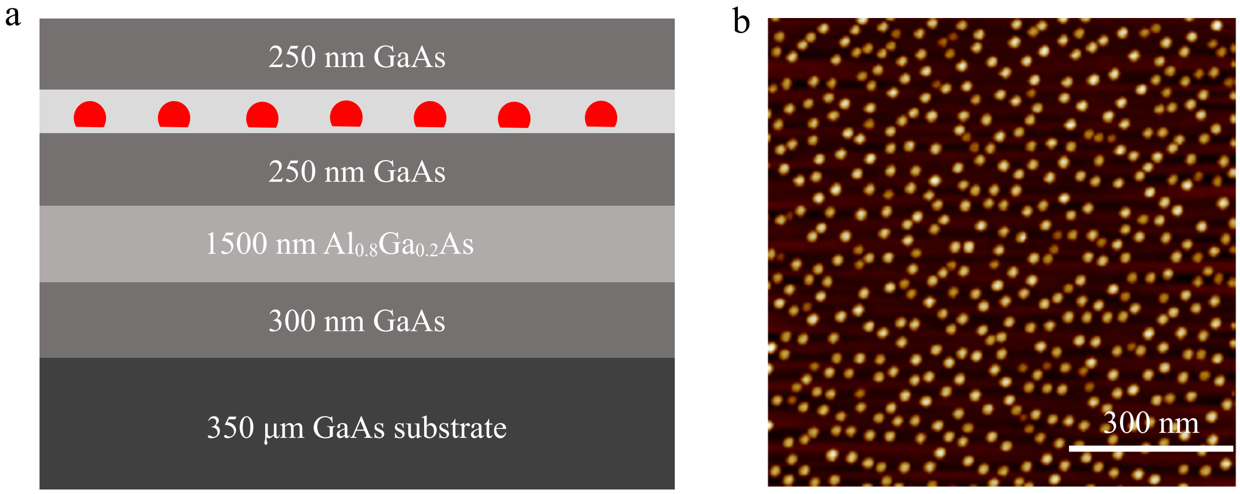

FIG. S1: **QD wafer.** a, Schematic epitaxial structure of wafer for mini-BIC laser. b, AFM image of the uncapped QDs.

II. DEVICE FABRICATION

The full fabrication flow of the mini-BIC laser is shown in Fig.S2. First, a layer of Si_3N_4 with a thickness of 200 nm was deposited on the wafer by inductively coupled plasma chemical vapor deposition (ICP-CVD) as a hard mask for PhC dry etching. A 400nm ARP6200 electron beam resist was spin coated on the surface of the hard mask. Subsequently, the electron beam lithography (EBL) was used to define the PhC pattern in ARP6200. The PhC pattern was transferred from resist into the hard mask layer using reactive ion etching (RIE). Afterwards, the electron beam resist was removed by Inductively coupled plasma etching (ICP) with O_2 plasma. Then a chloride-based etching was performed to obtain the air holes through the active layer. The residual Si_3N_4 hard mask was removed by RIE dry etching. Finally, the sacrificial layer was undercut by immersing the sample in 10% hydrofluoric acid solution for 1min30s to form a suspended PhC membrane.

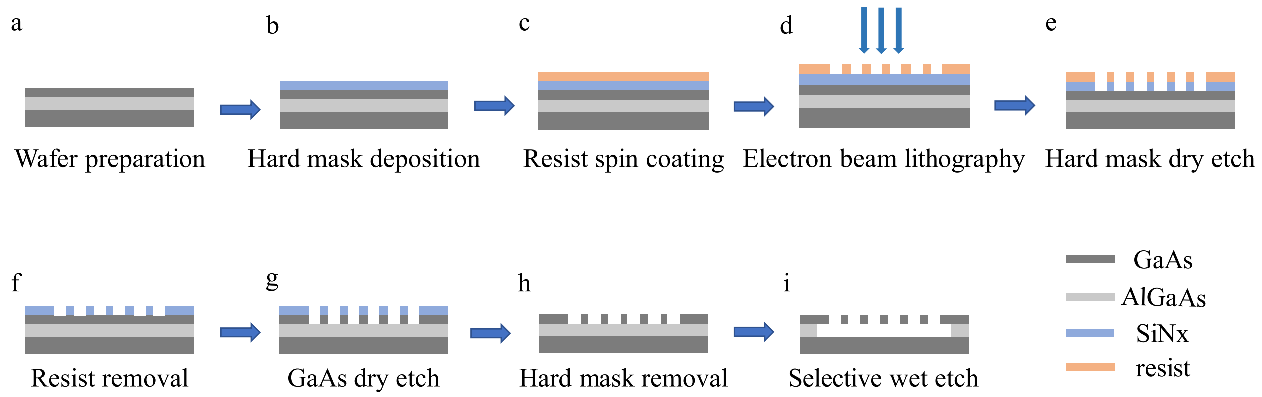

FIG. S2: **Fabrication flow of the mimi-BIC laser.** a, As-grown epitaxial wafer. b, Deposition of Si3N4 hard mask. c, Spin-coating of E-beam resist as a soft mask. d, Patter definition with EBL. e, Transferring PhC pattern to hard mask layer by RIE. f, Removal of the E-beam resist. g, Transferring pattern to active layer by ICP. h, Removal of the hard mask by RIE. i, Creation of the suspended membrane with a selective wet etching.

III. OPTICAL SETUP

The cuostomied confocal μPL setup for optical characterizations is shown in Fig. S3. The device is excited optically by a 780 nm CW laser diode or a pulse laser (10 ps, 86MHz period) through a microscope objective with a numerical aperture (NA) of 0.65. The spot size of the pump laser is about 8.11 μm, as shown in Fig. S4. The signal emitted from the sample is collected by the same objective, and then analyzed by a spectrometer (iHR550) with a resolution of 0.025 nm or guided to time correlated single-photon-counting (TCSPC) measurements for the second-order correlation or lifetime measurements via a flip mirror. For TCSPC measurements, the signal first passes through a home made grating filter with a spectral resolution of 1 nm to isolate the laser mode. Then the filtered signal went into either one superconducting nanowire single-photon detectors (SNSPD) for lifetime measurements or into a 50:50 fiber beam splitter (BS) and two SNSPDs for intensity autocorrelation measurements.

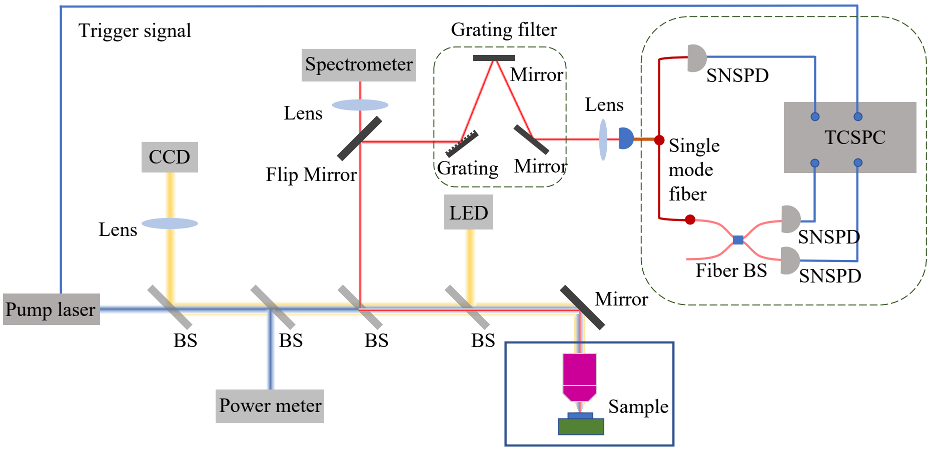

FIG. S3: **Experimental setup for Optical characterization.**

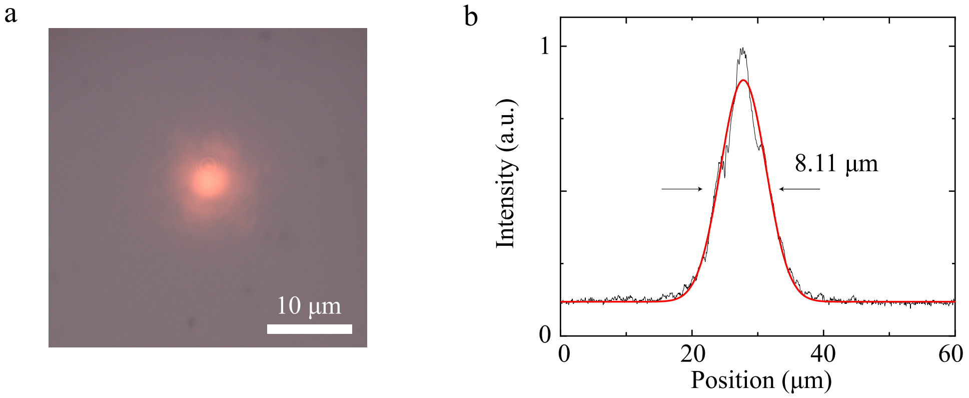

FIG. S4: **The estimate of the pump spot size.**, a, CCD (charge-coupled device) image of the pump spot. b, The 1D intensity profile fitted with a Gaussian curve (red line) .

IV. PL SPECTRA FOR CAVITIES WITH DIFFERENT PHC LATTICE CONSTANT A

In order to confirm that the measured sharp resonances are associated to the BICs in the PhCs. We systematically fabricated a serial of cavities with different lattice constant a. The PL spectra for the devices with different a are shown in Fig. S5. The cavity resonance shifts to the longer wavelength, following the change of the photonic bandgap.

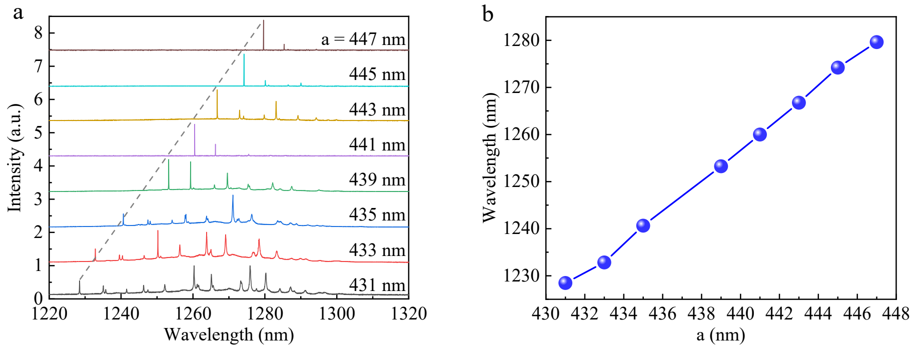

FIG. S5: **Tuning the cavity modes by varying the lattice constant a.** a, Spectra of the cavity modes for the photonic crystals with different lattice a. b, The optical resonant wavelength of the cavity mode M_{11} as a function of lattice constant a.

V. COMPARISON OF THE LASING AND NON-LASING DEVICES

The lasing devices is that the optical gain experienced by the lasing mode is significantly higher than those of other non-lasing modes, which facilitates the desirable single-mode lasing for applications. For the lasing device, the spectral distributions of the cavity modes respective to the gain spectrum is shown in Fig. S6(a), in which the lasing mode and non-lasing modes are distributed on one side of the gain peak to maximize difference of the optical gains experienced by the lasing mode and non-lasing modes. On the contrary, for "less optimal device" the high-Q mode and low-Q modes are spectrally distributed on both sides of the gain peak, which results in a smaller gain difference and consequently prevents the single-mode lasing, as shown in Fig. S6(b).

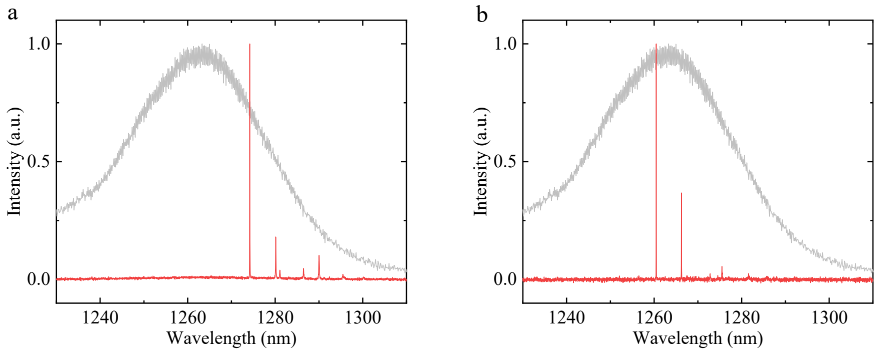

FIG. S6: **Comparison of the lasing and naon-LED devices.** a, Gain spectrum and the cavity modes for the lasing device. Mode M_{11} and M_{12} exhibit a large difference of the optical gain, which facilitate single-mode lasing operation. b, Gain spectrum and the cavity modes for the nano-LED device, preventing the lasing oscillation.

VI. PULSED EXCITATION

Fig. S7 presents the comparison of the lasing and non-lasing devices under pulsed excitation. The lasing device exhibited single-mode operation with typical lasing signatures of a nonlinear increased IO curve and a linewidth reduction behavior. Similarly, the nano-LED also showed a conventional "laser-like" behavior in both the IO curve and linewidth.

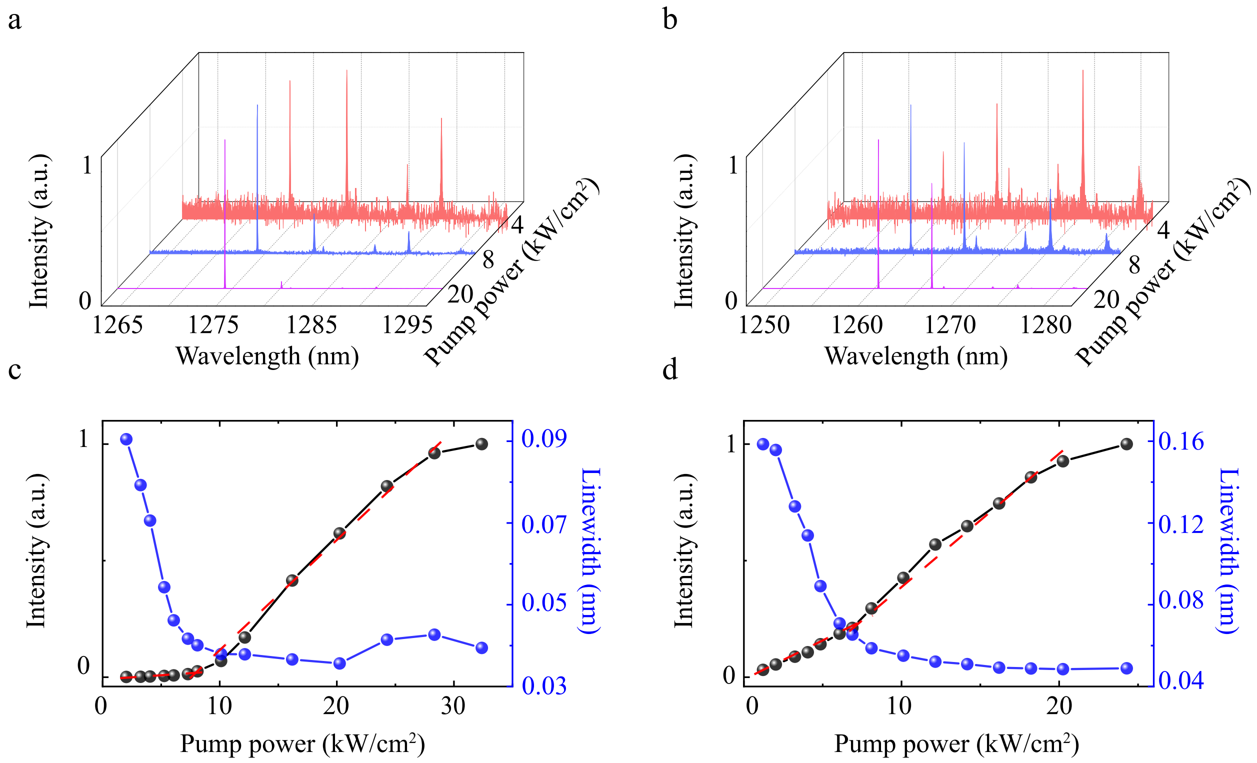

FIG. S7: **Characterizations of lasing and non-lasing devices under pulsed excitation.** a,b, Evolution of the normalized emission spectra of the lasing device (a) and non-lasing device (b) with the increased excitation power. c,d, Integrated output intensity and linewidth of the cavity mode M11 as a function of the excitation power, both showing "threshold-like" behaviors.

VII. COMPARION OF MINI-BIC LASER WITH OTHER BIC LASERS

TABLE S1: **Comparison of the mini-BIC laser with other BIC lasers.**

	Laser type	Pump method	Threshold peak power (mW)	Threshold power density (kW/cm^2)	Q factor
Nature 541, 196 (2017)	BIC	Pulse	15.6	$\sim$4	$\sim$4701
arXiv:1707.00181	BIC	Pulse	73	-	-
Nature Nanotech. 13 (2018)	BIC	Pulse	8.80×10ˆ5	7.0×10ˆ4	2750
npj 2D Materials and Applications.3 (2019)	BIC	CW	-	0.144	2500
Science 367 (2020)	BIC	Pulse	5.28×10ˆ5	4.2×10ˆ4	-
Nano Lett. 20 (2020)	BIC	Pulse	5.09×10ˆ8	1.8×10ˆ5	2590
arXiv:2012.15642	BIC	Pulse	$\sim$2.16×10ˆ6	$\sim$2.75×10ˆ8	$\sim$2883
Nature Commun. 12 (2021)	Super-BIC	Pulse	0.34	1.47	$\sim$7300
This work	Mini-BIC	CW	0.041	0.08	$\sim$32500
